# Physiological-social score (PMEWS) vs. CURB-65 to triage pandemic influenza: a comparative validation study using community-acquired pneumonia as a proxy

**DOI:** 10.1186/1472-6963-7-33

**Published:** 2007-03-01

**Authors:** Kirsty Challen, John Bright, Andrew Bentley, Darren Walter

**Affiliations:** 1 Emergency Medicine and Planning, University Hospital of South Manchester NHS Foundation Trust, Manchester, UK; 2 Respiratory Medicine, University Hospital of South Manchester NHS Foundation Trust, Manchester, UK; 3 Respiratory Medicine and Critical Care, University Hospital of South Manchester NHS Foundation Trust, Manchester, UK; 4 Emergency Medicine, University Hospital of South Manchester NHS Foundation Trust, Manchester, UK

## Abstract

**Background:**

An influenza pandemic may increase Emergency Department attendance 7-fold. In the absence of a validated "flu score" to assess severity and assist triage decisions from primary into secondary care, current UK draft management recommendations have suggested the use of CURB-65 and chest X-ray as a proxy. We developed the Pandemic Medical Early Warning Score (PMEWS) to track and triage flu patients, taking into account physiological and social factors and without requiring laboratory or radiology services.

**Methods:**

Validation of the PMEWS score against an unselected group of patients presenting and admitted to an urban UK teaching hospital with community acquired pneumonia. Comparison of PMEWS performance against CURB-65 for three outcome measures: need for admission, admission to high dependency or intensive care, and inpatient mortality using area under ROC curve (AUROC) and the Hanley-McNeil method of comparison.

**Results:**

PMEWS was a better predictor of need for admission (AUROC 0.944) and need of higher level of care (AUROC 0.83) compared with CURB-65 (AUROCs 0.881 and 0.640 respectively) but was not as good a predictor of subsequent inpatient mortality (AUROC 0.663).

**Conclusion:**

Although further validation against other disease datasets as a proxy for pandemic flu is required, we show that PMEWS is rapidly applicable for triage of large numbers of flu patients to self-care, hospital admission or HDU/ICU care. It is scalable to reflect changing admission thresholds that will occur during a pandemic.

## Background

As part of planning for a potential H5N1 influenza pandemic, using United Kingdom Department of Health and Health Protection Agency projections[1], we have been forced to acknowledge that our urban Emergency Department, which normally sees approximately 250 patients per day, will potentially see an additional 1500 attenders per day with influenza-like illness and associated anxiety at a pandemic peak. The potential magnitude of the "worried well" attendance is supported by the experience of the Dutch Municipal Health Service during the H7N7 avian outbreak in 2003 where of 453 patients presenting to a screening centre only 109 were serologically positive for influenza[2].

In the absence of diagnostic criteria, and a validated influenza "severity scoring system", current draft plans use instruments designed for the assessment of pneumonia severity as a proxy tool. Examples include the British Thoracic Society's CURB-65, the American Thoracic Society guidelines[3] and the Pneumonia Severity Index[4]. These have been validated as predictors of mortality in a population with community acquired pneumonia[5] and include either radiological or laboratory investigations. This renders them cumbersome and mandates hospital-based assessment for a decision on the need for admission or discharge.

We designed a simple and rapidly applicable purely clinical scoring system for use in primary and secondary care and at other points of contact with health providers in the community. This tool is not intended to be diagnostic, as during a pandemic all patients with influenza-like illness will be assumed to have pandemic influenza[6]. Our aim was to create a screening tool for adults to identify the need for hospital admission and importantly to reassure and discharge those who do not require hospitalisation at the point of assessment. We suggest the ideal score should reflect acute physiological derangement but also consider and accommodate age, chronic disease co-morbidities and other social factors. It will be necessary for such a scoring system to be used serially to triage and determine the need for admission and for higher levels of care[7] and even possibly predict mortality. When applied across a health economy, it should provide consistency and reassurance to those patients who seek alternative opinions from a number of sources.

**Figure 1:**
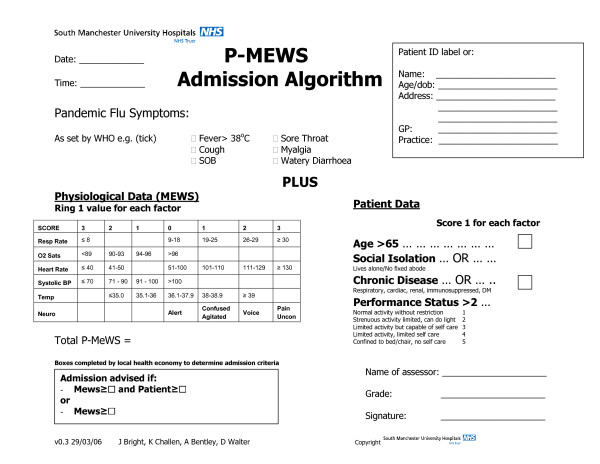
PMEWS algorithm.

We therefore modified our hospital Medical Early Warning Score[8] to include transcutaneous oxygen saturation and added supplementary scoring features of co-morbidity and social factors to influence the threshold for making admission and discharge decisions (Pandemic Medical Early Warning Score; PMEWS). Our score incorporates an extra point for age ≥ 65 years and another for any of a) social isolation (defined as living alone or having no fixed abode), b) chronic disease (respiratory, cardiac, renal, diabetes mellitus or immunosuppression of any cause) or c) performance status of limited activity or worse (modified Karnofsky >2[9]). Fig 1 summarises the full score with symptomatology to be defined by World Health Organisation as disease evolution takes place.

We describe the first validation of the PMEWS scoring system in an unselected population of patients with community acquired pneumonia (as a proxy for pandemic influenza) presenting and admitted to our urban teaching hospital from February to December 2005. We compared it with the current UK standard for community-acquired pneumonia, the CURB-65 score, which has also been recommended as an instrument for triage in the event of an influenza pandemic[1].

## Methods

The University Hospital of South Manchester NHS Foundation Trust is an 895-bed urban teaching hospital providing secondary care services to a population of 500,000 and tertiary care in burns and plastic surgery, cardiology, cardiothoracic surgery, maxillofacial surgery, vascular surgery and respiratory medicine. The Emergency Department sees 78000 new attendances per year both by Primary Care referral and from self-attendance. An Early Warning Score was, at the time of data collection, used by the nursing staff to identify higher-risk patients but this and CURB-65 were used for admission decision-making only at the doctors' discretion.

The study population consisted of a) all adult (> 15 years old) patients presenting to the Emergency Department who were deemed by the Emergency Physician to have pneumonia and b) all adult patients admitted to the hospital whose discharge diagnostic code included ICD-10 J18 (pneumonia) and where non-aspiration pneumonia was the working diagnosis at first consultant contact following admission. Patients were excluded if, on review of the case notes, insufficient data was present to calculate a PMEWS or CURB-65 score, or if it was apparent from the case notes that the pneumonia was hospital-acquired, or that the reason for admission was another diagnosis. Immunocompromised patients were specifically not excluded as immune status is often unknown at presentation and previous research has demonstrated mixed results in terms of the effect of immunosuppression on outcome[10,11]. South Manchester Local Research Ethics Committee waived the need for formal ethical approval as no additional direct patient contact was involved.

The study population was a retrospectively identified cohort for the period February – December 2005 using the Emergency Department local coding system and the hospital Patient Administration System (IBA inc). Contemporaneous data was collected to calculate the PMEWS and CURB-65 scores using the Emergency Department nursing and medical records and the admitting physician clerking. Where data varied, the earliest recorded information was used. If data on one or two variables was missing this was assumed to be normal. If data on three or more was missing the patient was excluded.

The reference standards, to reflect the clinically significant decision points, were a) admission to hospital on the day of examination, b) admission to the intensive care (ITU) or high dependency (HDU) units or the use of non-invasive ventilation at any time during the admission, c) mortality during in-patient stay.

Data was analysed using SPSS 11.5 (SPSS inc^®^) to calculate ROC curves. The z-statistic for comparison between ROC curves was calculated using the method described by Hanley and McNeil[12], with a z-value of >1.96 considered significant at the 95% confidence level.

## Results

**Figure 2:**
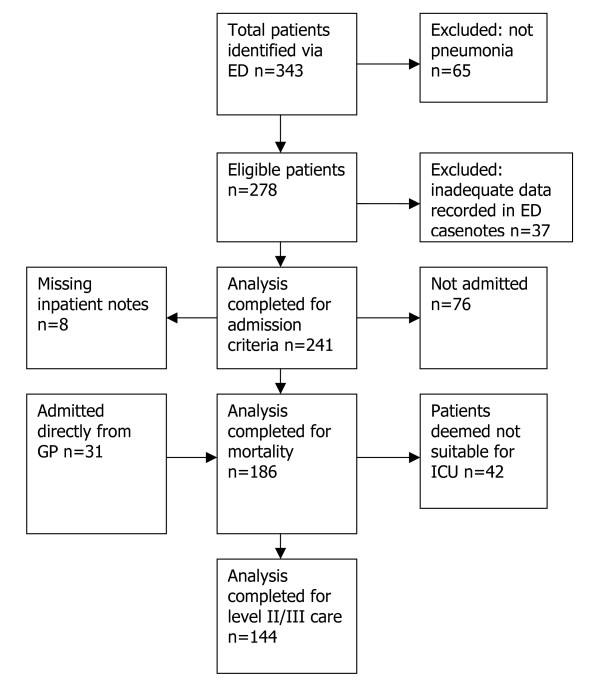
Cohort diagram.

Patients identified from February – December 2005 are shown in Fig 2. 31 patients referred directly from general practice to inpatient teams for admission were not treated by the Emergency Department and have therefore not been included in the analysis of admission/discharge decisions. 37 ED patients had insufficient data in the casenotes to complete either CURB-65 or MEWS scores and were therefore pragmatically excluded. Baseline demographics are shown in table 1. 194 patients were admitted of which 8 sets of notes were unavailable. 42 received HDU or ITU care (23 intubated and ventilated, 12 non-invasively ventilated) and a further 42 were deemed inappropriate for HDU/ITU care due to pre-existing comorbidities or performance status or to patient preference. 42 patients died.

**Table 1:** Demographic characteristics

**Decision to admit analysis (n = 242)**	
**Age ≥ 65**	127 (52.5%)
**Socially isolated**	75 (31.0%)
**Relevant comorbidities**	102 (42.1%)
**Performance status ≥ 3**	82 (33.9%)

**Level II/III care and mortality analysis (n = 186)**	

**Age ≥ 65**	131 (70.4%)
**Relevant comorbidities**	60 (32.3%)
**Performance status ≥ 3**	93 (50%)

**Figure 3:**
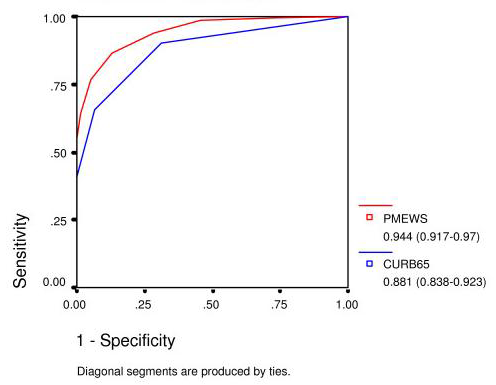
ROC curve for admission decision.

**Figure 4:**
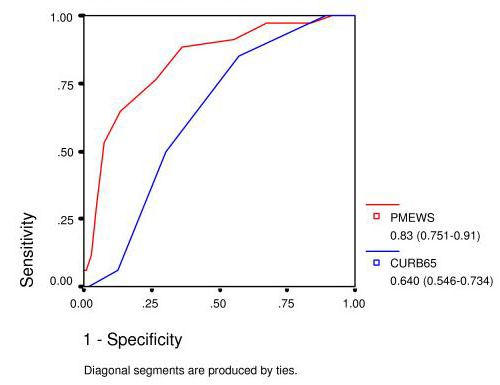
ROC curve for admission to level 2 or 3 care.

**Figure 5:**
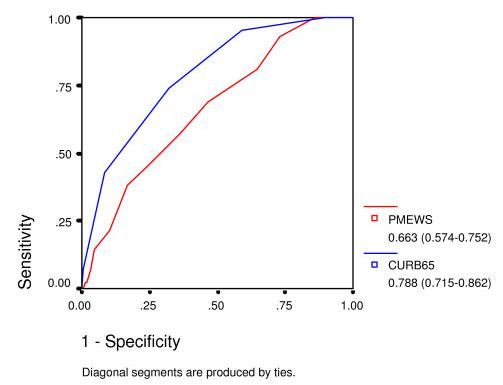
ROC curve for mortality.

Table 2 shows the three outcome measures stratified by CURB-65 and PMEWS scores. Table 3 shows predictive values for various cut-off points for each of the scores. Figs 3, 4, 5 show ROC curves with AUROC and 95% confidence intervals for each of the outcome measures. Once admitted to hospital, the social factor becomes irrelevant and is not included as a discriminator for higher level care or mortality. Calculations for HDU/ITU care exclude those patients for whom it was deemed inappropriate.

**Table 2:** Outcome measures by CURB-65 and PMEWS score

		Outcome 1: Admission n = 241		Outcome 2: ICU (level 3) or HDU (level 2) care n = 144		Outcome 3: Mortality n = 186	
		
		Admitted	Not admitted	Level 2/3 care	Level 0/1 care	Death	Survival
CURB-65	0	16	53	0	12	0	15
	1	40	19	5	35	2	44
	2	42	5	12	30	9	39
	3	39	0	15	19	13	34
	4	23	0	2	12	15	11
	5	4	0	0	2	3	1

PMEWS	0	1	20	0	2	0	2
	1	1	22	0	7	0	7
	2	8	13	1	9	0	11
	3	12	12	0	18	3	19
	4	16	6	2	13	5	12
	5	20	3	1	21	5	26
	6	15	1	4	11	5	15
	7	24	0	4	14	5	17
	8	14	0	4	7	3	11
	9	16	0	8	3	7	9
	10	13	0	6	2	3	8
	11	9	0	1	1	3	2
	12	5	0	1	1	2	2
	13	3	0	0	1	0	1
	14	2	0	1	0	1	1
	15	1	0	0	0	0	0
	17	1	0	1	0	0	1
	24	1	0	0	0	0	0

	NR	2	0				

NR: not recorded

**Table 3:** Predictive value of scores

	**Score**	**Sensitivity**	**Specificity**	**PPV**	**NPV**
Outcome: admission	CURB-65 ≥ 2	65	93	95	56
	CURB-65 ≥ 3	40	100	100	44
	PMEWS>1	98	55	82	95
	PMEWS>2	93	71	87	84
	PMEWS>3	86	87	93	75
	PMEWS>4	76	94	96	65
	PMEWS>5	64	98	99	56
	PMEWS>7	40	100	100	44
	PMEWS>9	21	100	100	37
	PMEWS>11	8	100	100	34

Outcome: HDU/ITU admission	CURB-65 ≥ 3	50	70	34	81
	PMEWS>3	97	32	30	97
	PMEWS>4	91	45	34	94
	PMEWS>5	88	64	43	95
	PMEWS>6	76	74	47	91
	PMEWS>7	64	86	59	89
	PMEWS>9	29	95	67	81
	PMEWS>11	8	98	60	78

PPV: positive predictive value

NPV: negative predictive value

The z-statistic as described by Hanley and McNeil is 3.09 for admission decision, 3.76 for admission to HDU/ITU care, and 2.48 for mortality.

## Discussion

The PMEWS score performs well for discerning those patients in need of admission (area under ROC curve 0.944) and triages effectively those in need of higher levels of care before irreversible organ dysfunction is established (area under ROC curve 0.83), whereas CURB-65 achieves comparable areas under the ROC curve of only 0.81 and 0.64 respectively. PMEWS is particularly strong in the mid-range of values where we hypothesise those patients who are most likely to benefit from intensive treatment lie. It provides quick and simple but robust information and could be applied consistently at the Emergency Department, in the General Practice surgery, at proposed Community "Flu Centres" and even by District Nurses and the Ambulance Service paramedics and Emergency Care Practitioners seeing patients in their own homes. CURB-65, in comparison, requires laboratory investigation and, as has been noted elsewhere, is not particularly effective at identifying patients requiring higher levels of care[13]. If CRB-65 is employed by removing the need for laboratory investigations as has been suggested in the BTS guidelines, this score is further weakened in its ability to identify patients requiring higher levels of care. It has also been noted that CURB-65 may be misleadingly low in fit young adults with certain atypical pneumonias[14].

CURB-65 remains a strong tool for the prognostication of mortality, the task for which it was originally designed. The PMEWS score was designed from a generic early warning score as an isolated rapid "snapshot" tool which relies on physiological derangement at the time of assessment for triage to appropriate levels of care. A single physiological assessment at admission cannot be expected to perform well as a mortality predictor over the course of an entire hospital admission. We note that our mortality rate (at 22.5%) is higher than previous reports of community-acquired pneumonia but suggest that this is due to our pragmatic inclusive strategy; Fine, for example, excluded patients from nursing homes[11]. The mean age of our patients, at 70.9 years is notably higher than that studied by Kamath et al, which was 58.8 years[15], and 70% of our patients were aged 65 or over, compared with 58% of Lim et al's group[16].

National predictions on the incidence of a pandemic influenza[1], together with the inevitable complication of the "worried well" seeking reassurance, suggest an increase in Emergency Department attendance of 700%, mandating a new approach to triage and assessment of patients with influenza related illness. While clinical intuition may be the mainstay of assessment, an objective and effective decision tool applied consistently across a health economy should instil confidence both in the decision makers and the patient population and may reduce the level of "second opinion" seeking. There will need to be empowerment of a wide variety of healthcare professionals to make decisions about management and destination to manage the patient surge and we believe that an objective scoring system based on simple physiology is appropriate. It is not feasible, at least within our institution, that our pathology or radiology services will be able to perform in the region of 900 extra serum urea analyses or chest X-rays per day. Although concerns have recently been expressed about the practical applicability of an early warning score during disease outbreaks[17], the concept has a precedent in Critical Care Outreach for determining need for higher levels of care amongst a general ward population. Early Warning Scores (EWS) are already in widespread use in hospitals throughout the UK. They are tools familiar to many health care providers whereas CURB-65 may have a more limited appeal and familiarity[18].

It is likely that in a pandemic thresholds for hospital admission will be higher than is current practice. While it is impractical prospectively to validate a pandemic score in the absence of a pandemic, we believe that the performance of PMEWS in identifying patients currently admitted to higher-intensity care demonstrates its scalability for greater numbers. Whatever the nature of the illness, decisions relating to the need for hospital admission relate to the physiological state and the potential for further deterioration where intervention may limit or prevent this. In the face of overwhelming demand, the threshold for therapeutic intervention and admission could be altered by raising the decision score required within PMEWS, as demonstrated in table 2. This "degradation of care with scale" as demand outstrips supply is a recognised part of the need to do the most for the largest number of patients and would not be achievable within the relatively limited confines of CURB-65.

No disease-related scoring system can confidently be stated at present to be appropriate for application to pandemic influenza since the actual nature of the disease is unknown. Although we present here validation against a relatively small population with community-acquired pneumonia, further validation using wider patient sets (including SARS, sepsis and unselected ED presentations) is ongoing. Our patient physiology and social circumstances based score could be used as an admission decision tool, whatever the nature of the final presentation of the next pandemic of influenza. As the numbers rise, knowledge of the course of the disease becomes more established and capacity of hospitals declines, then there is capacity for scaling the referral to hospital/admission criteria by adjusting the score threshold for referral. We believe that, when adopted across a health economy, the adjustable threshold of PMEWS will greatly enhance the decision value of the system, compared with the relative inflexibility of a scoring system such as CURB-65.

## Conclusion

Although no scoring system can be fully validated for an influenza pandemic given that the pattern of the disease is currently unknown, we have demonstrated, using community-acquired pneumonia as a proxy, that a non-disease-specific physiological-social score performs as effectively to triage patients to appropriate levels of care as a disease-specific score.

## Competing interests

The author(s) declare that they have no competing interests.

## Authors' contributions

All authors contributed to the design of the score and to drafting the final manuscript. KC and JB carried out data collection. KC carried out statistical analysis with advice from AB and DW. All authors read and approved the final manuscript.

## Appendix 1

CURB-65 score

One point for each of:

**C**onfusion

**U**rea >7 mmol/l

**R**espiratory rate >= 30/min

low systolic (<90 mmHg) or diastolic (<= 60 mmHg) **B**lood pressure

age >= **65 **years

British Thoracic Society. Guidelines for the management of community acquired pneumonia in adults – 2004 update. London: British Thoracic Society, 2004.

## Pre-publication history

The pre-publication history for this paper can be accessed here:


